# Context matters for primary health care access: a multi-method comparative study of contextual influences on health service access arrangements across models of primary health care

**DOI:** 10.1186/s12939-018-0788-y

**Published:** 2018-06-15

**Authors:** Bernadette Ward, Riki Lane, Julie McDonald, Gawaine Powell-Davies, Jeff Fuller, Sarah Dennis, Rachael Kearns, Grant Russell

**Affiliations:** 1 School of Rural Health, Monash University, PO Box 666, Bendigo, VIC Australia; 2 Department of General Practice, School of Primary and Allied Health Care, Monash University, Clayton, Australia; 3 Centre for Primary Health Care and Equity, University of New South Wales, Sydney, Australia; 4 College of Nursing & Health Sciences, Flinders University, South, Bedford Park, South Australia; 5 Faculty of Health Sciences, The University of Sydney, Lidcombe, Australia; 6 South Western Sydney Local Health District, Ingham Institute of Applied Medical Research, Liverpool, Australia

**Keywords:** Access, Equity, Primary care, Models, Policy, Governance, Context

## Abstract

**Background:**

Equitable access to primary health care (PHC) is an important component of integrated chronic disease management. Whilst context is known to influence access to PHC, it is poorly researched. The aim of this study was to determine the contextual influences associated with access arrangements in four Australian models of integrated PHC.

**Methods:**

A multi-method comparative case study design. Purposive sampling identified four models of PHC across six sites in two Australian states. Complexity theory informed the choice of contextual factors that influenced access arrangements, which were analysed across five dimensions: availability and accommodation, affordability, acceptability, appropriateness and approachability. Semi-structured interviews, document/website analysis and non-participant observation were used to collect data from clinicians, administrative staff and other key stakeholders. Within and cross-case thematic analysis identified interactions between context and access across sites.

**Results:**

Overall, financial viability, objectives of the PHC model and relationships with the local hospital network (LHN) underpinned access arrangements. Local supply of general practitioners and financial viability were strong influences on availability of after-hours services. Influences on affordability were difficult to determine because all models had nil/low out-of-pocket costs for general practitioner services. The biggest influence on acceptability was the goal/objectives of the PHC model. Appropriateness and to a lesser degree affordability arrangements were influenced by the relationship with the LHN. The provision of regular outreach services was strongly influenced by perceived population need, referral networks and model objectives.

**Conclusions:**

These findings provide valuable insights for policy makers charged with improving access arrangements in PHC services. A financially sustainable service underpins attempts to improve access that meets the needs of the service population. Smaller services may lack infrastructure and capacity, suggesting there may be a minimum size for enhancing access. Access arrangements may be facilitated by aligning the objectives between PHC, LHN and other stakeholder models. While some access arrangements are relatively easy to modify, improving resource intensive (e.g. acceptability) access arrangements for vulnerable and/or chronic disease populations will require federal and state policy levers with input from primary health networks and LHNs.

## Background

Internationally, strong primary health care (PHC) systems are associated with improved health outcomes for people with chronic diseases [1]. In response to the increasing burden of chronic disease [2] and evidence of inequitable access to health care [3], many high-income countries are developing services with a focus on the pillars of PHC: accessible, comprehensive, continuous, first-contact care [4]. While all of these pillars are important, access is core as it is the point where services meet the population and their health needs. This is important for those with relatively high health care needs; particularly those with chronic disease.

Arrangements to improve health system supply of accessible care have been linked to improvements in population health. PHC researchers, practitioners, policymakers and stakeholders in 14 different countries have identified similarities in the setting, population group and funding of interventions targeted at improving organisations’ ability to supply accessible care [5]. A recent scoping review found that organisational interventions aimed at improving approachability, availability and affordability of PHC services could improve population health outcomes [6]. While access factors are broadly known to be applicable across national health systems, the impact of contextual influences on access arrangements is poorly researched [7]. These include, national (e.g. health system regulations and funding), local (e.g. workforce supply and socio-economic status) and practice-level (e.g. service history and staffing arrangements) influences [8]. Internationally, understanding these associations may assist health systems to learn from each other to achieve the associated population health outcomes.

Efforts to address poor access to PHC services also need to take into account context-related factors. In recent decades, the Australian healthcare system has been characterised by a range of state and federal funding initiatives for integrated PHC approaches (models). That is, these provide person-focused population-based care that is continuous, comprehensive and coordinated across the health service [9]. These models vary in noteworthy aspects [10], and so provide a ‘natural experiment’ to examine how contextual factors influence the enactment of initiatives aimed at improving services’ ability to supply accessible care. Within the Australian healthcare system these PHC models are characterised by some similar constraints (e.g. fee-for-service (FFS) arrangements). However, they are sufficiently different to enable comparisons and establish an evidence-base to improve PHC access. While there is a plethora of literature on ‘accessible’ health care services, research evidence exploring the role of context is critical for policy makers/practice leaders charged with improving access to PHC [11].

Australia has a complex public/private mix for both primary and hospital-based care. PHC services typically include general practitioners (GPs) working in private practice and to varying degrees, other health professionals, including nurses and allied health professionals. In addition to these mainstream PHC services, there are other PHC services designed specifically to meet the needs of particular populations. These include (but are not limited to) Aboriginal Community Controlled Health Organisations (ACCHOs), migrant and refugee health services, prison health services, sexual health services. The universal health system is FFS and remunerates GPs, specialist doctors and limited services from some allied health staff, often with client co-payments. In addition, incentive payments for nurses and priority target activities (e.g. chronic disease management plans) are funded at the federal level; separately from state/territory funded community health services and public hospitals. Public hospital services are free at the point of delivery, but often have substantial waiting lists. They are grouped into Local Hospital Networks (LHNs) (also known as Local Health Networks, Local Health Districts, Hospital and Health Services, and Metropolitan Health Services), which provide in-patient, out-patient and off-site medical, nursing and allied health services, including co-location in PHC. LHNs play significant roles in community health services but these vary between states. Private health insurance does not cover medical services in PHC but partially covers additional costs in private hospitals and allied health, which often have reduced waiting lists [12].

The increasing burden of chronic disease and escalating costs of delivering primary health care has led to calls to improve the accessibility of primary health care services [13]. The aim of this study was to determine how contextual factors influence access (availability and accommodation, affordability, acceptability, appropriateness and approachability) goals and arrangements, in four Australian PHC models. We sought to provide useful insights for policymakers seeking to identify transferable characteristics of PHC models that will reduce inequitable access to PHC services.

## Methods

### Study design

A multi-method comparative case study design was used [14]. Online lists were used to develop a sampling frame of multidisciplinary co-located PHC models (involving at least three different professional groups including GPs, nurses and allied health) in three Australian states. Purposeful sampling for maximum variation [15] was used to select cases (PHC services) that included a range of medical, nursing and allied health services, sizes, locations, and policy supports.

Eleven co-located and multidisciplinary PHC services were invited to participate via a letter/email or telephone call to the practice manager. Representatives of four different PHC models (traditional GP practices, GP Super Clinic (GPSC), HealthOne service and a Community Health Service) expressed interest in participating. A brief summary of these PHC model types and objectives is provided in Table 1.

**Table 1:** Types and objectives of PHC models in the study

Model	Objectives
Traditional GP practice	Traditional GP practices are typically privately owned by one or more GPs and generally include practice nurses. They may also include allied health and other visiting specialist staff. There are no explicit published objectives available for this model.
GP Super Clinic (GPSC)	GPSCs were introduced by the federal government in 2010 [25]. The broad objective of these services was to provide accessible integrated multidisciplinary care through physical or virtual co-location.
HealthOne	HealthOne services were established by the New South Wales state government in 2006/07 [26] with the aim of providing integrated, client focused, multidisciplinary team care, with stronger links between general practice and state funded community health services.
Community Health Service	The Community Health Service (CHS) program was introduced by the federal government in the early 1970s. Of relevance to the Victorian CHS in this study, the Victorian state government reorganised CHSs in the late 1980s with the broad aim of providing universal access to services, largely through non-government organisations with community based boards; particularly for vulnerable populations.

HealthOne and Community Health Services are state models in which public funding allows no or minimal patient co-payments for GP, nurse or allied health services

### Conceptual frameworks

The Levesque et al., [16] framework defines access dimensions (availability and accommodation, affordability, acceptability, appropriateness and approachability) which informed the assessment of access goals and arrangements. Table 2 provides examples of each of these dimensions of access that are relevant to health service supply. These examples are not comprehensive, and were selected as illustrative based on analysis of the measures available in this study.

**Table 2:** Access dimension definitions [16] and supply examples

Access dimension	Examples
*Availability and accommodation*	
“Health services (either the physical space or those working in health care roles) can be reached both physically and in a timely manner.”	Onsite after-hours (AH) (i.e. after 6 pm weekdays; weekend opening) Same day/walk-in GP appointments
*Affordability*	
“The economic capacity for people to spend resources and time to use appropriate services.”	Size of patient co-payments for GPs and for other co-located services.
*Acceptability*	
“Cultural and social factors determining the possibility for people to accept the aspects of the service.”	Having dedicated culturally safe and appropriate services
*Appropriateness*	
“The fit between services and clients need, its timeliness, the amount of care spent in assessing health problems and determining the correct treatment and the technical and interpersonal quality of the services provided.”	Co-location of allied health professionals and medical specialists Comprehensive assessment and case/ care management
*Approachability*	
“People facing health needs can actually identify that some form of services exists, can be reached, and have an impact on the health of the individual.”	Outreach (e.g. home/residential aged care facility visits) and other programs

The complexity theory analytic framework by Miller et al., [8] was used to examine context. This included:

- *history and initial conditions,* e.g. history of the service, funding arrangements, business model, practice priorities regarding access;
- *agents,* e.g. staffing arrangements (total number of equivalent full-time (EFT) of all staff, governance and stability (management and staffing);
- *local fitness landscape,* e.g. location (Australian Standard Geographical Classification – Remoteness Area (ASGC-RA)) [17], catchment profile including Index of Relative Socio-economic Advantage and Disadvantage (IRSAD) score [18], identified GP district of workforce shortage [19], relationship with local services such as the LHN;
- *regional and global influences,* e.g. larger health care system, culture and regulations.

Complexity theory is widely used in health services research to examine interactions and how this might influence change [20].

### Data collection

Data were collected in 2015 via semi-structured interviews, document (e.g. procedure and information technology manuals, organisation charts) and website analysis, and non-participant observation during a three-day visit to each site and a series of follow-up telephone calls/emails over a three-month period. Representatives from each clinician group, administrative staff (executive and management, reception, other administration staff), and other local stakeholders (e.g. other health care providers) were invited to participate in 20–40 min semi-structured initial and follow-up interviews, which included questions about access and context, at a mutually convenient time and location. Each interview was recorded using a digital recorder and transcribed verbatim. Non-participant observations of the immediate physical environment, front desk, administrative staff procedures and practices were recorded as field notes as were notes from document analysis and researcher reflections. The ULTRA Practice Environment Template [21] was used to guide the non-participant context observation data collection. This validated tool accounts for the variation between medical practice environments [22].

### Data analysis

The qualitative and quantitative data were collected concurrently and analysed within and across cases [14]. The qualitative transcripts, document analysis, field notes, non-participation observation records and ULTRA site description templates were managed and thematically coded in NVivo 10 [23]. Two researchers read and coded the transcripts to develop the preliminary coding framework. The coding framework and ‘thick’ description summaries assisted with identifying the need for additional data collection. Feedback to each case was undertaken as a form of member checking [24]. This resulted in minor changes to the practice environment data in three settings. A matrix was developed for each case to examine the interactions between context and access. Cross-case comparisons (using coded qualitative and quantitative data) were used to relate similarities and differences to site characteristics and other contextual factors [14]. Quantitative data were used to describe cases and access arrangements.

Ethical approval was granted by the Hunter New England Local Health District, Western NSW Local Health District, Monash Health, UNSW and Monash University Human Research Ethics Committees. Interviewees provided written consent to participate and PHC cases were offered $1000 for their involvement in data collection.

## Results

Six multidisciplinary and co-located PHC Services (an extended GP practice, three GPSCs, a HealthOne and a Community Health Service) participated. As summarised in Table 3, PHC models and their patient populations varied. As per Table 4, 88 staff participated in semi-structured interviews (17 GPs, 24 nurses, 20 allied health providers, 17 administration staff and 10 others). The analysis of these and other qualitative data are presented in tables and text that describe the contextual factors, access goals and arrangements for each case.

**Table 3:** Summary of participating cases’ context factors (history and initial conditions and local fitness landscape)

GP 1: Urban general practice, set up and owned by principal GP. It evolved from an existing practice. Patient profile includes older regular patients and newer younger families who have moved into the area. The catchment and patient population includes a small proportion of indigenous and CALD (culturally and linguistically diverse) people. GPSC 2: Outer urban service under the federal government GPSC program. It is a newly established service on the grounds of a university. Many services linked with the LHN. The patient profile includes a mix of older regular patients, younger families, a significant university student population and a particular focus on people (including adolescents) with mental health conditions. The catchment and patient population includes a small proportion of indigenous and CALD people. GPSC 3: A regional two-site service established under the federal government GPSC program. Site A is relatively new and the service bought the established general practice (site B). A few services are linked with the LHN. The patient profile includes younger people who commute to the city for work, AH walk in patients from out of area or other practices (site A), older regular patients and retirees (site B), and some indigenous and/or CALD people. GPSC 4: A regional (urban) service established under the federal government GPSC program. It evolved from an established general practice. Many services are linked with the LHN. The patient profile includes regular patients, AH non-regular and patients who use only the visiting specialist services. The practice provides services to refugees from one particular ethnic group and to client groups that other practices in the area are reluctant to take on, such as alcohol and other drug users and residents in aged care facilities. HealthOne (HO) 5: A rural practice under the state government HealthOne program. It evolved from an existing service. The patient profile includes older local residents and those living in outlying towns, as well as patients with chronic and complex conditions especially targeted by the service. CHS 6: An urban practice, part of a recent merger of several community health services under the state government community health program. The patient profile includes predominantly people from socially disadvantaged backgrounds, including a large CALD population. As there is a large refugee, asylum-seeker and recent migrant population, they provide a refugee health assessment service.

**Table 4:** Number of interviews at each service by disciplinary background

Service	GPs	Nurses	Allied health	Admin	Other^a^	Total
GP 1	3	5	2	3	4	17
GPSC 2	2	1	5	3	2	13
GPSC 3	2	6	2	4	0	14
GPSC 4	4	3	2	2	2	13
HO 5	2	5	5	3	2	17
CHS 6	4	4	4	2	0	14
Total	17	24	20	17	10	88

^a^ Medical specialist, external provider/agency

**Fig. 1:**
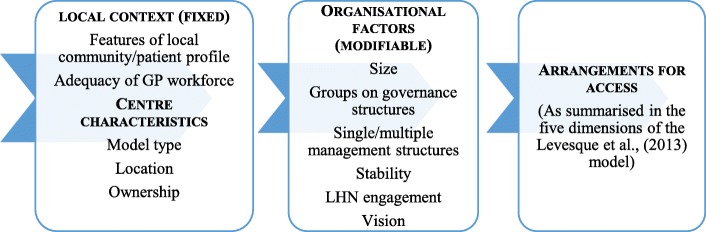
Access to primary health care logic model

Findings are reported in accordance with the logic model in Fig. 1. The assumption underlying the model is that arrangements for ensuring access (as summarised in the five dimensions of the Levesque et al., [16] model) are influenced fixed and modifiable factors. That is, aspects of local context are essentially fixed (e.g. the model under which they operate) while some organisational and/or functioning are modifiable (e.g. size, representation on governance structures).

Within and across sites, to varying degrees, access arrangements were influenced by national health system policies, model objectives, practice size, local relationships and perceived population need. Concerns about the financial viability of the practice influenced the degree to which arrangements were made to improve some aspects of access (e.g. acceptability for vulnerable populations).

### Contextual factors

Table 5 provides a more detailed overview of the participating cases’ contextual factors. The cases varied in business model, size, workforce availability, governance, stability, location and relationship with LHN.

**Table 5:** Summary of contextual factors by caseDomain and description

	Case and PHC model					
	1 - Traditional	2 - GPSC	3 - GPSC	4 - GPSC	5 - HealthOne	6 - Community Health
*History and Initial Conditions*						
Funding arrangements (All cases had access to FFS via GPs and other eligible services, and to nurse incentive payments)	Rental from co-located services	Rental from co-located services, other grants	Other grants	Rental from co-located services, other grants	Rental from co-located services, other grants	Broad range of grants
Business model (FP – for-profit; NFP-not-for-profit; PPP-public/private partnership)	FP (privately owned)	NFP	FP (privately owned)	PPP (university, hospital, LHN)	PPP	NFP
*Agents*						
Size (no. of equivalent full-time (EFT) staff) (small ≤ 20; medium ≥ 21 < 35; large ≥ 35)	Small	Medium	Large	Large	Medium	Large
GPs (no. of EFT)	5 GPs, EFT 3.5	5 GPs, EFT 2	12 GPs, EFT 4	23 GPs, EFT 7	5GPs EFT 3	9GPs, EFT 5.4
Workforce (District of Workforce Shortage) (Changes to GP workforce availability in last 5 years)	No (recent increase -oversupply)	No (well serviced)	Yes (shortage)	No (recent increase - oversupply)	Yes (shortage)	No (oversupply)
Governance (Board of Management representation: e.g. GP/LHN/PHC network and/or university representative/s)	GPs only	University, GPs	CEO is owner and primary decision maker	LHN, University	LHN, PHC network, GPs	Independent board members; external to case LHN, LGA, other managers
Stability (Recent changes to structure, governance, workforce)	Instability (workforce leadership)	Stable	Stable	Instability (management and governance structures)	Stable	Instability (management structures)
*Local fitness landscape*						
ASGC-RA Remoteness index (Major City; Inner Regional; Outer Regional)	Major City	Major City	Inner Regional	Inner Regional	Inner & Outer Regional	Major City
Local government area (LGA) population [41]	64,000	25,000	76,000	100,000	13,000	107,000
IRSAD^a^ (LGA): Decile	5	8	9	6	7	9 (practice population has lower socio economic status)
Links with LHN/acute health services (distance to acute hospital, co-located specialist clinics/community health services)	< 20kms, none	< 1 km, many LHN clinics	Site A < 28 km, Site B < 12 km, no LHN clinics	< 500 m, some LHN clinics	< 1 km, most community health services	< 5kms, one LHN clinic
*Regional and global influences*						
Service culture (referral to other providers within the service, relationship with LHN staff)	Strong informal referral culture	Strong referral networks in and across services	Communications in and across service have improved	Instability has made ‘whole of service’ culture difficult	Strong referral networks in and across services	Allied health and medical siloes impair communication

^a^ Index of Relative Socio-Economic Advantage and Disadvantage IRSAD: (based on LGA). The lowest 10% of areas are given a decile of 1 and the highest 10% a decile of 10

Only the smallest case (1) did not have a broad range of co-located allied health professionals. Co-location of other health professionals in all cases provided patients with access to on-site allied health and, in some, medical specialist health services. Cases 2, 3 and 4 had a strong focus on appropriateness via a broad range of medical specialists, while case 6 had only one, and the two smaller cases (1,5) had none. Case 5 and 6 had a strong focus on acceptability through attention to cultural variability while case 2 had an emphasis on approachability via the provision of mental health services. The larger cases with governance and/or management instability) also had problems with service culture; particularly with relationships between disciplines within the service (case 6), or with the LHN (case 4).

### Influence of context factors on access vision and goals

Vision statements typically give direction to employees and so are closely linked with goals. Consistent with their model objectives, three of the federal or state government cases (GPSC, HealthOne and CHS) had explicit goals to improve access [25–27]. This took the form of enhancing access for specific population groups. Staff from most cases referred to a “one-stop shop” and “everything under one roof”.

The understanding and sharing of vision across staff differed according to the PHC model, business model, size, establishment history and presence of co-located health professionals. The relatively new cases (2,3,4) had very different visions to that of case 6, which focused on providing services to marginalised communities. In contrast to the smaller traditional ‘bottom-up’ practice (case 1), these new cases were less likely to have a shared understanding of vision. The same was also true for the larger cases where there were more part-time staff and sessional co-located staff than the medium and smaller cases. An underlying theme across most cases (especially 1,2,3 and 4) was the business model needed to maintain financial viability and GP income. Hence, emphasis on some access arrangements within the overall vision was often associated with local factors (e.g. perceived local population need for services) that promoted financial viability. These included extending AH services in response to the local commuting population (case 3), and improving organisational (e.g. billing) systems (case 1 and 2).

### Influence of context factors on access arrangements

All cases provided on-site access to primary medical care (GPs and nurses) and allied health professionals, with 2,3,4 and 6 also providing access to specialist medical care. The range varied from two/three (case 1) through to co-location of a much broader range of allied health disciplines, and multidisciplinary services such as mental health, drug and alcohol (cases 2,3,5 and 6).

Some cases placed greater emphasis on different factors influencing access that responded to local context (see Table 6). For example, cases 3,4 and 5 emphasised provision of AH GP services, which increased availability and accommodation. This was particularly important for case 3, located in a workforce shortage area (see Table 5). Similarly, while case 6 had no AH provision, affordability (no/low co-payments for all of its services) was important as they prioritised providing services to vulnerable populations (see Table 5).

**Table 6:** Summary of access arrangements by case

Access dimension and example/s	Case and access arrangements					
	1	2	3	4	5	6
*Availability & accommodation*						
Onsite AH GP services (i.e. after 6 pm weekdays; weekend opening)	No AH	Some AH	Good AH	Good AH	Good AH	No AH
Same day/walk-in GP appointments available	Yes	Yes	Sometimes	Yes	Yes	Sometimes
*Affordability*						
Patient co-payments for other co-located services, which may vary across allied health/medical specialist and public/private (All had nil/low co-payments for GP services)	Medium	Low/medium	Low/medium	Low/medium	No/low/medium	No/low
*Acceptability*						
Unique responses to acceptability to fit with context	Nil	Dedicated youth mental health	Indigenous sensitive with Aboriginal nurse	Reception area not welcoming, ad-hoc arrangements for practitioners seeing vulnerable populations	Information customised to literacy, cultural variability	High use of interpreters and information in other languages, gender diversity sensitive practices, well known as service for vulnerable populations
*Appropriateness*						
Co-location of allied health/medical (med) specialists (public(pub)/private(priv))/ LHN clinics	Few allied health (priv), No med special or LHN	Good range allied health &med special (priv/pub), LHN clinics	Good range allied health (priv), some med special (priv), no LHN	Range of allied health & med special (pub/priv), LHN	Broad range allied health (pub), no med specialist, LHN	Good range allied health (pub), few med special, one LHN
*Approachability*						
Outreach programs (all provide some services in residential aged care settings and home visits)	Ad hoc (schools, community events)	Regular mental health clinic and school services	Nil extra	Nil extra	Regular community groups and surrounding town clinics	Dedicated staff who do outreach in a range of settings

Access arrangements were related to a range of contextual factors, in particular: PHC model, local workforce supply conditions, service stability, identified local need, relationship with the LHN and financial viability (see Table 7). While the PHC model objectives were linked to availability and accommodation, participants reported that local under or over supply of GPs (closely linked with perceived population need) and financial viability were strong influences on the availability of allied health services. Influences on affordability were difficult to determine; all cases had nil/low co-payments for GP services, and the vast range of allied health/specialist services meant that client co-payments were often set at the discretion of individual practitioners. The availability of these via the LHN improved affordability. The policy objectives of case 6 meant that all services were low/no out-of-pocket costs. This service was in an area where the population had high levels of socioeconomic advantage; however, their patient profile was one of socioeconomic disadvantage. While identified need and financial viability was associated with improved acceptability, the biggest influence on these acceptability arrangements was model objectives. Appropriateness arrangements were strongly influenced by the relationship with the LHN (e.g. represented in governance arrangements, co-location of LHN staff). The provision of regular unique outreach services (approachability) was strongly influenced by identified need, referral networks and model objectives.

**Table 7:** Unique influence of context factors on increase/decrease in access arrangements and subsequent service level

Access dimension and example/s	Context factors and unique influence on access arrangements (increase ↑, decrease↓ service level)								
	Objectives of PHC model	Case size (small, medium, large)	Local workforce supply market conditions	Service stability (recent changes to structure, governance)	Location (ASGC)	Local population/patient profile identified need	IRSAD (local pop^n^, or patient profile)	Relationship with LHN	Financial viability
*Availability & accommodation*									
Onsite AH i.e. after 6 pm weekdays; on weekends	Where explicit↑	Small↓	Shortage/oversupply of GPs↑,↓	Stable structure, governance and leadership may↑	Inner/Outer regional↑	Identified need↑	No clear association	No clear association	Focus on financial viability↑,↓
Same day/walk-in GP appointments	Where explicit↑	No clear association	No clear association	No clear association	No clear association	No clear association	No clear association	No clear association	No clear association
*Affordability*									
Patient co-payments for non GP co-located services	Where explicit for vulnerable (including children) populations↑	No clear association	No clear association	No clear association	No clear association	Identified need↑	Ad hoc arrangement↑,↓	Strong presence of LHN services associated with ↑ in affordability	Focus on financial viability↑,↓
*Acceptability*									
Unique responses to acceptability to fit with context	Where explicit for vulnerable populations↑	No clear association	No clear association	Governance and leadership stability ↑	No clear association	Identified need↑	No clear association	No clear association	Focus on financial viability (sub-population opportunity) may↑
*Appropriateness*									
Co-location of allied health professionals (allied health)/medical specialists (med spec – priv/public)/Local hospital network (LHN) clinics	No clear association	No clear association	No clear association	No clear association	No clear association	No clear association	No clear association	Strong LHN relationship, referral network and communication↑	Focus on financial viability (via rental opportunity) may↑
*Approachability*									
Outreach programs (all provide some services in residential aged care settings and home visits)	Where explicit for vulnerable populations↑	No clear association	No clear association	No clear association	No clear association	Identified need↑	No clear association	No clear association	Focus on financial viability (sub-population opportunity) may↑

## Discussion

Access is one of the key characteristics of high-performing PHC services [28]. This study of four models of PHC across six cases has shown how contextual factors influenced access arrangements (availability and accommodation, affordability, acceptability, appropriateness and approachability). While access arrangements varied between and within the PHC models, as part of their standard operations, all cases made access arrangements. This is consistent with the international focus on improving organisations’ capacity to supply accessible care [5]. Our findings indicate there are some overarching non-mutually exclusive context drivers that, to varying degrees, influence access arrangements within a service. These include financial viability, model objectives, and LHN/other stakeholder objectives.

Access arrangements were improved when financial viability was underpinned by capitation style funding models and not totally reliant on FFS funding. This is consistent with the findings of a Canadian study of PHC models and is possibly linked to the broad context of Australia’s universal health scheme regulations [29]. In England, PHC services with higher levels of funding have been associated with better patient outcomes [30]. However, this weighted, capitated funding model is not used in Australia so GP funding is reliant on patient throughput. Hence, total reliance on a FFS funding stream may limit access arrangements; the financial viability of extending GP AH/walk-in services (availability and accommodation), may be compromised. The cases in this study where participants described high levels of acceptability had dedicated funding to provide services to vulnerable populations. The provision of dedicated services with high levels of acceptability can be resource intensive in terms of staff costs, infrastructure and community relationships [31]. This is consistent with our finding that smaller services lacked the infrastructure, economies-of-scale and capacity of medium and larger services to diversify their funding sources and provide dedicated services for vulnerable populations.

The objectives of the different PHC models appeared to influence access arrangements. However, the PHC models with a focus on providing services to vulnerable populations were underpinned by capitation style funding. Unsurprisingly, these cases had best acceptability arrangements. This is consistent with the findings of an Australian case study of GPSCs where program objectives were most likely to be achieved when financial/infrastructure support was provided [22]. In this study, the best availability arrangements (for AH services) were in cases where availability and accommodation was a priority and the geographical area was poorly serviced AH by GPs; this meant that providing AH services was financially viable. In North America, patient-centred medical homes have been associated with improved access arrangements (particularly those that are more difficult to modify such as acceptability and appropriateness) [32]. Service models that prioritise population need and have financial ‘flexibility’ are more likely to innovate and develop effective team-based PHC; another of the key characteristics of high-performing PHC [28]. This is consistent with reports from a Canadian study that examined differences in access between new and traditional models of PHC [33]. Compared to the traditional PHC models, the new models of care derived significantly less funding from traditional GP funding streams, were more likely to provide AH services, team care and were associated with patients’ reports of higher levels of appropriateness and acceptability [33]. While the underlying business model (e.g. for-profit or other) also influences financial viability, Australian NFP and PPP PHC services are also expected to be financial viable [34]. Some dimensions of access arrangements are more difficult to alter according to context, so servicing these sub-populations needs to be explicit in model objectives and reflected in funding agreements.

Access arrangements may be facilitated by the coalescence of PHC and LHN/other stakeholder model objectives. Appropriateness objectives via co-location of LHN specialist outreach clinics may serve LHN objectives (e.g. to reduce their waiting lists), but also improve access arrangements within PHC (e.g. affordability) [22]. Similarly, university allied health student clinics may increase access to PHC care whilst also meeting clinical placement needs. In this study, these were linked to strong PHC staff vision, relationships with the LHN/university staff (e.g. via governance representation) and stable PHC structure, governance and workforce arrangements.

Access arrangements that are comparatively easy to modify may be optimised by finding a ‘fit’ between model, context and stakeholder objectives. However, these need to be examined via the lens of financial viability and sources of funding and accountability (e.g. federal versus state services). There is a range of policy-amenable interventions that can be implemented to improve access to PHC, but these will require use of policy levers by federal and State/Territory governments. Adopting a greater population health and equity orientation for PHC would require further reforms including appropriately directed funding. Suggested approaches include: alternative payment mechanisms such as those seen in patient-centred approaches [35–37]; public/private partnership models involving LHNs; and outreach and network models. Involving LHNs through co-location of allied health and medical specialist services can provide patients with convenient access to LHN services, more affordable alternatives, and models of care that target and address the needs of hard to reach populations in the catchment area.

### Study limitations

We used methods that are typical of comparative case studies. The detailed descriptions, within and subsequent cross-case thematic analysis and interpretations enhance the validity of the findings [38]. However, the study had several limitations. The focus was on multidisciplinary models of Australian PHC, but logistical constraints limited the sample size and range of PHC models. The number of qualitative interviews was relatively large (*n* = 88) but were limited to staff from six PHC services, which may limit the transferability of the findings. We did not include an ACCHO; these PHC services are initiated and operated by local Aboriginal communities and aim to deliver holistic and culturally appropriate care. Given this, our findings cannot necessarily apply to ACCHOs or other models, such as hub and spoke or telehealth. We focused on the supply of access to health services and so did not investigate the paired patterns of consumer demand for access. While there is a plethora of research on the association between the social determinants of health and consumer demand for access to health services [39, 40], further research is needed to study the impact and outcomes of supply arrangements and how they influence consumers’ PHC access experiences and health outcomes within a broader range of PHC models.

## Conclusion

Strong PHC systems are associated with improved population health outcomes [1]. In order to improve the supply of PHC, services need to be accessible and equitable [3]. Increasingly, PHC models include a focus on improving access but the role of context in influencing health service access arrangements is poorly understood. The findings of this study of four PHC models indicate funding arrangements that support financial viability, model objectives and relationships with the LHN are the strongest influences on access arrangements. While some access arrangements are relatively easy to modify, improving access (e.g. acceptability) for vulnerable and/or chronic disease populations will require federal and state policy levers with input from primary health networks and LHNs.

## Abbreviations

AH: After-hours
ASGC-RA: Australian Standard Geographical Classification – Remoteness Area
CALD: Culturally and linguistically diverse
EFT: Equivalent full-time
FFS: Fee-for-service
FP: For-profit
GP: General practitioner
GPSC: General practice super clinic
IRSAD: Index of Relative Socio-economic Advantage and Disadvantage
LGA: Local government area
LHN: Local hospital network (including Local Health Network, Local Health District, Hospital and Health Service, Metropolitan Health Service etc)
NFP: Not-for-profit
PHC: Primary health care
PPP: Public private partnership
